# Six stormy years and the audacity to confront a challenging future: taking stock of the Kenyan Doctors’ Union

**DOI:** 10.11604/pamj.2018.31.31.16153

**Published:** 2018-09-13

**Authors:** Aruyaru Stanley Mwenda, Alex Muturi, Fredrick Ouma Oluga

**Affiliations:** 1 Department of Surgery, Consolata Hospital Nyeri, Kenya; 2 Department of Surgery, Tigoni District Hospital, Limuru-Kenya; 3 School of Medicine, University of Nairobi, Kenya; 4 Secretary General, Kenya Medical Practitioners, Pharmacists and Dentists Union (KMPDU), Nairobi-Kenya

**Keywords:** Human resources for health, doctors´ union, advocacy, health service commission, industrial unrest

## Abstract

The Kenya Medical Practitioners, Pharmacists and Dentists Union (KMPDU) was formed in August 2011. Within the last six years, this union has galvanized the Kenyan doctors together, agitated for healthcare policy reforms and successfully negotiated and registered a Collective Bargaining Agreement (CBA). Though political will and the national citizens' psyche on matters pertaining to public healthcare remain a challenge, this union has made its foot prints on the Kenyan conversation space. KMPDU looks forward to engaging local, regional and international health stake holders to improve the state of the country's health care, key among these being to have a national commission handling all the human resources for health.

## Opinion

In 2011, after a rigorous and tedious process, the Kenya Medical Practitioners, Pharmacists and Dentists Union (KMPDU) was registered [1]. This was made possible after promulgation of Kenya's new constitution which has a comprehensive bill of rights that accords every worker the right to join and participate in the activities of a trade union [1, 2]. This union was born at a time when myriad challenges faced the health sector-poor staffing, poor remuneration, challenges with post graduate training and inadequate health care funding from the government [1]. Though the primary role of a trade union is members' welfare mostly in matters remuneration, the doctors' plight has been impossible to dismember from the patients'. As such, the union has seen its mandate drift from mere agitation for its members' rights and remuneration to essentially health policy advocacy. In this article, we look through the milestones since the formation of the union, the challenges that persist and the promise ahead.

**Formation of the union:** The union was duly registered in August 2011 following years of lobbying and mobilization [1]. Since its inception, it has galvanized the voice of the Kenyan doctor on matters health advocacy and doctors' welfare.

**Milestones:** It may look sadistic to enlist industrial actions and demonstrations as milestones, but this is one area every Kenyan citizen would easily remember about KMPDU in recent days. Numerous industrial actions have taken place since the registration of the union with the latest one being a 100-day strike that saw union leaders imprisoned for contempt of court and harassed by political and social leaders [1, 3, 4]. What many might not know is that the doctors took these steps as the last option when dialogue with government authorities was no longer bearing fruits. Looking at the positive side of things, this series of strikes is what has kept the government on its toes regarding making their pledges and promises about health remain on course. Another great stride has to do with uniting the doctors and helping in bonding the union members together. Usually there will exist a gap between consultants and junior doctors, with much of their interaction happening at the work place. During the successive strikes and other national mobilization events, doctors have managed to bridge these gaps by coming together during branch meetings, nationwide doctors' assemblies and shared social medial groups. Often branch officials (more junior) have delegated some duties to consultants and professors all to a better member harmony. After the recent strike was called off, the doctors organized a medical camp and corporate social responsibility event where they spent a day at one of the Kenyan prisons, seeing the sick and donating foodstuff among other items. One could not hesitate to notice the ease with which the administration of this correctional facility handled the doctors. And the happiness on the faces of the inmates [5]. Just after the union turned 6, the Collective Bargaining Agreement (CBA) between the doctors and the 47 county governments was signed and deposited with the courts. This is after that between KMPDU and the national government and two other parastatals was signed immediately after calling off of the latest doctors' strike earlier in the year [6].

**Challenges:** The greatest challenge bedeviling the country's health sector at the moment is the devolution of most of the healthcare in accordance with the Kenyan constitution. Although certain county governments have made significant investment in health, the handling of the human resources for health has been a big headache. Different counties have been at loggerheads with the doctors because of complaints about transfers, release for post graduate training, prompt salary payments and insufficient staffing among others. It is the authors' believe, and in fact majority of Kenyan healthcare workers hopes that the urgent solution to healthcare human resources challenges at the moment will be the formation of a health service commission to handle all the human resources for health from a central national point. Even as we write, the nurses are on strike for the fourth month running. Clinical officers have joined them a week ago. Public health care remains nearly grounded. And this portends bad for the future of the country's health sector. The other challenge is the ignorant skeptical Kenyan. Him that will not differentiate between a doctor and the hospital cleaner wearing a lab coat. Her that will just accept and move on when the public hospital closes down. Him that does not bother to ask their elected leaders when hospitals run without adequate staffing or drugs. There is a degree of silence and apathy that even 100 days of doctors' strike and a week in jail could not uproot from majority of Kenyans. And majority of these are plainly ignorant. The middle-class Kenyan will not know that a hospital is understaffed and the few healthcare workers overworked. They had rather complain that the doctor is perpetually absent. They will not demand how come the taxes they pay cannot upgrade their local district hospital to match the services offered at the smaller private facility across the street. And finally, the political leader, who rarely seeks treatment from a public hospital will not bother to listen about public health facilities' concerns. The most we have gained has been task force reports and recommendations that end up being shelved off and forgotten. Having state officers compelled to seek treatment from public hospitals (at least the amenity wings) as long as their care is being catered for from the exchequer might be the intervention that saves our public health system. If recent outbreaks of cholera in our country are anything to go by, we are losing ground on issues we had taken care of a while back.

### The promise that lies ahead

**Influencing health related politics:** KMPDU is often invited to and also convenes key policy and implementation forums both at the county and the national level. Recently during the inauguration of newly elected governors, at least 5 counties recognized and invited KMPDU officials at the Branch level as part of their important guests. Additionally, the KMPDU has built a relevant relationship with several stakeholders in the public arena including the presidency of the country which would easily facilitate influence public policy on health off politics and through politics. The KMPDU sees a necessary need to engage directly with likeminded parliamentarians to influence healthcare policy. Finally, the KMPDU's active and vibrant membership is actively engaged in shaping public opinion through writings in the dailies, TV interviews hence driving the larger KMPDU positions and influence politics.

**Reverting human resources for health to the National government:** The Devolution of Health created enormous opportunities for faster and more region and/or disease burden specific decision making. However, devolution of health was carried out without a devolution policy. Devolution therefore created darkness within the health sector with little or no social and leadership accountability, uncoordinated policy development and implementation framework and incoherence in health planning and investments. Devolution of Health in Kenya must be revisited in order to develop a policy structure upon which financial, accountability and management clarity to citizens, sector actors and health professionals is achieved. The debate to revert healthcare management back to the national government has been alive since the health devolution in 2013. KMPDU holds the view that standardizing the aspect of Human Resources for Health management portends enormous benefits to strengthen the Health System and cure the challenges facing counties and the national government. One means to standardize is to create a Health Services Commission. The commission equally can handle several other aspects of health systems regulation and standardization such health data and information custody, infrastructure planning amongst others. To this extent, management of healthcare benefits from economies of scale and economies of impact on programs if there is a coordinated and concerted nationwide approach rather than a piecemeal county driven approach. This is an agenda item KMPDU shall engage Kenyans on for 2 years to 2019.

**Members welfare and health advocacy:** The welfare of doctors and health professionals in general is not separable from the general welfare of the patients from a quality perspective. KMPDU thus approaches advocacy for improvement of members' welfare by advocating for the general improvement and strengthening of the healthcare system. KMPDU advocates for increasing healthcare funding towards the Abuja Declaration of 2001 [7], reforming the legislative and regulatory framework for hospitals, medical insurance and health professionals that led to the enactment of the Health Act 2016 [8] and the improvement of quality of medical care. In the next five years KMPDU plans to campaign on a Universal Health Agenda to improve access, affordability, and quality of healthcare including promotion of healthy lifestyles. This advocacy shall include engaging relevant stakeholders to change necessary laws that hinder healthcare provision in any way. To this extent, KMPDU is already conducting an audit of all the laws, regulations and policies in the healthcare for purposes of streamlining them towards enabling a more efficient, accessible and affordable health service provision to all Kenyans. At the international level, the admission of KMPDU into the International Association of Medical Regulatory authorities (IAMRA) accords the platform for these ideas and agenda items to be spread beyond the country's borders.

## Conclusion

Six years after its formation, KMPDU has covered significant milestones given the prevailing environment. The biggest challenge remains in winning the political opinion leaders and arousing the interest in health matters among the Kenyan citizens. We hope in the next 5 years; the union should be able to shape policy on matters healthcare reform and reorganization of the human resources for health through the creation of a central body to handle this challenging duty.

## Competing interests

The authors declare no competing interest.
